# Hepatic pseudolymphoma with hepatobiliary-phase ring-like hyperintensity on gadoxetic acid–enhanced MRI: radiologic–pathologic correlation with intratumoral fibrosis

**DOI:** 10.1093/bjrcr/uaag021

**Published:** 2026-05-21

**Authors:** Shuji Nagata, Nona Fujimoto, Tatsuyuki Tonan, Tomonori Chikasue, Akiko Sumi, Jun Akiba, Shuichi Tanoue

**Affiliations:** Department of Radiology, Kurume University School of Medicine, Kurume, Fukuoka, 830-0011, Japan; Department of Radiology, Kurume University School of Medicine, Kurume, Fukuoka, 830-0011, Japan; Department of Radiology, Kurume University Medical Centre, Kurume, Fukuoka, 839-0863, Japan; Department of Radiology, Kurume University School of Medicine, Kurume, Fukuoka, 830-0011, Japan; Department of Radiology, Kurume University School of Medicine, Kurume, Fukuoka, 830-0011, Japan; Department of Pathology, Kurume University School of Medicine, Kurume, Fukuoka, 830-0011, Japan; Department of Radiology, Kurume University School of Medicine, Kurume, Fukuoka, 830-0011, Japan

**Keywords:** Hepatic pseudolymphoma, reactive lymphoid hyperplasia, MRI, gadoxetic acid-enhanced MRI, hepatobiliary phase, hepatobiliary-phase ring-like hyperintensity, intratumoral fibrosis

## Abstract

Hepatic pseudolymphoma (HPL) is a rare benign lymphoproliferative disorder that can mimic malignant hepatic tumors on imaging. Hepatic pseudolymphoma is usually small and solitary; however, multiple lesions can develop in some cases. Herein, we report multiple HPLs with an unusual hepatobiliary finding on gadoxetic acid-enhanced MRI. A woman in her 50 s with non-alcoholic fatty liver disease was incidentally found to have 2 hepatic tumors: a 3-cm lesion in segment 5 (S5) and a 1-cm lesion in segment 8 (S8) during pre-operative cholelithiasis evaluation. The laboratory tests, including liver function and tumor marker levels, were unremarkable. The 2 lesions were hyperintense on fat-suppressed T2-weighted images and showed restricted diffusion. On dynamic gadoxetic acid-enhanced MRI, both lesions demonstrated faint arterial enhancement and hepatobiliary-phase hypointensity, with peritumoral arterial-phase hyperenhancement. Additionally, the S5 lesion showed faint linear hyperintensity adjacent to the lesion on diffusion-weighted imaging and contained an internal ring-like area that was hypointense on T2-weighted images but hyperintense within an otherwise hypointense lesion in the hepatobiliary phase. As malignancy could not be excluded, laparoscopic anterior sectionectomy was performed. Histopathological examination confirmed reactive lymphoid hyperplasia in both lesions. In the S5 lesion, the intratumoral fibrotic tissues formed a ring-like structure corresponding to the hepatobiliary-phase hyperintensity. Hepatobiliary-phase ring-like hyperintensity attributable to intratumoral fibrosis in HPL has not been previously reported. Awareness of this imaging–pathology correlation may improve interpretation of HPL imaging and help differentiate it from malignant hepatic tumors that can show hepatobiliary-phase hyperintensity, including hepatocellular carcinoma, cholangiocarcinoma, and metastases.

## Clinical presentation

A woman in her 50 s initially visited a referring hospital due to biliary colic. She was then diagnosed with cholelithiasis. During pre-operative imaging evaluation, hepatic tumors measuring 3 cm were identified in segment 5 (S5) and 1 cm in segment 8 (S8) of the liver, prompting referral for further assessment. Her medical history was significant for non-alcoholic fatty liver disease. She had no relevant family history and no history of smoking or alcohol consumption.

## Investigations/imaging findings

Based on the laboratory examinations, the patient had normal liver function parameters, including aspartate aminotransferase (22 U/L) and alanine aminotransferase (30 U/L) levels. The tumor marker levels, including PIVKA-II (35 mAU/mL), carcinoembryonic antigen (0.5 ng/mL), and carbohydrate antigen 19-9 (2.9 U/mL), were within normal limits. The serologic tests for hepatitis B virus surface antigen and antibody, hepatitis C virus antibody, and human T-cell leukemia virus type I/II yielded negative results. No other significant abnormalities were detected.

**Figure 1:**
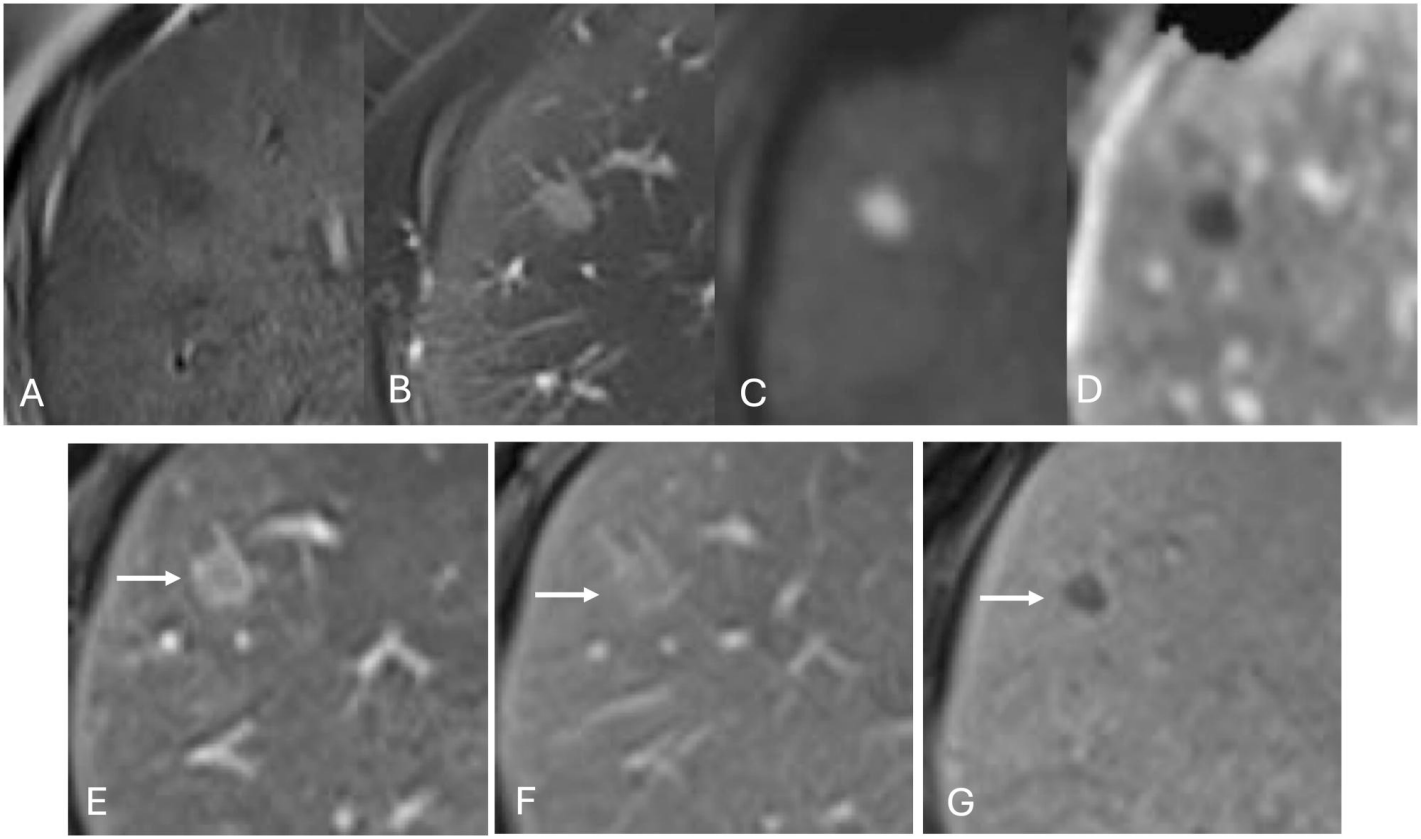
(A-C) A lesion in S8 showed hypointense on a T1-weighted image (A) and hyperintense on a fat-suppressed T2-weighted image (B) and diffusion-weighted imaging (C). (D) The ADC map of the tumor indicated an ADC value of 0.85 × 10^−3^ mm^2^/s. (E-G) On gadoxetic acid-enhanced (EOB) MRI, the lesion showed a faint enhancement in the arterial phase (E), appeared isointense relative to the surrounding liver parenchyma in the portal phase (F), and showed hypointense in the hepatobiliary phase (G). The peri-tumoral regions demonstrated early enhancement in the arterial phase (arrow in E), with persistent enhancement in the portal phase (arrow in F), and showed isointense to the surrounding liver parenchyma in the hepatobiliary phase (arrow in G).

**Figure 2:**
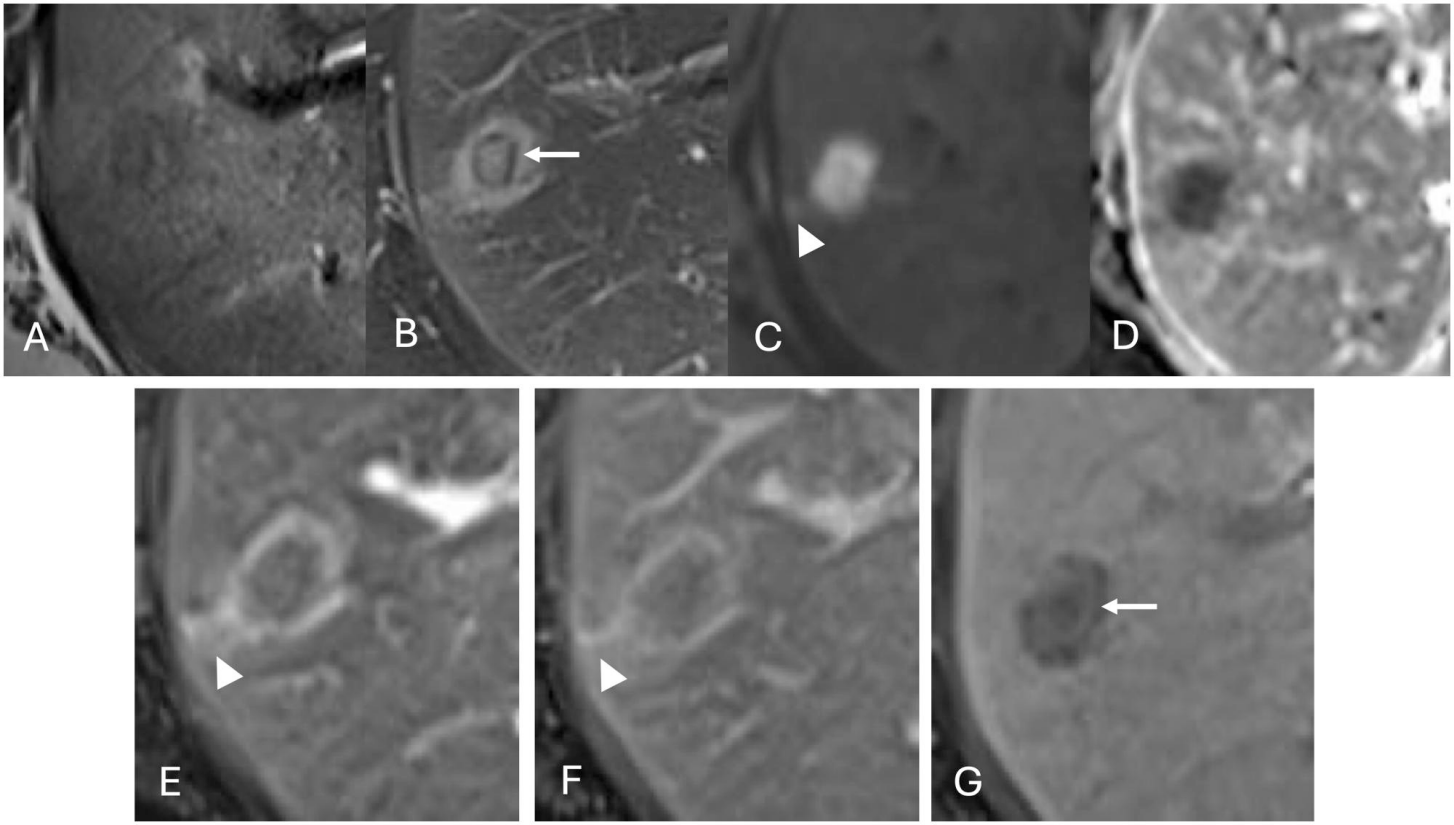
(A-C) A lesion in S5 showed hypointense on a T1-weighted image (A) and hyperintense on a fat-suppressed T2-weighted image (B) and diffusion-weighted imaging (C). The lesion contained an internal ring-like area that appeared hypointense on T2-weighted images (arow in B). (D) The ADC map of the tumor indicated an ADC value of 0.65 × 10^−3^ mm^2^/s. (E-G) On gadoxetic acid-enhanced (EOB) MRI, the lesion showed a faint enhancement in the arterial phase (E), appeared isointense relative to the surrounding liver parenchyma in the portal phase (F), and showed hypointense in the hepatobiliary phase (G). The peri-tumoral regions demonstrated early enhancement in the arterial phase (E) with persistent enhancement in the portal phase (F) and were shown to be isointense to the surrounding liver parenchyma in the hepatobiliary phase (G). There was linear hyperintensity close to the lesion on diffusion-weighted imaging (arrowhead in C) and enhancement in the arterial phase (arrowhead in E) and portal phase (arrowhead in F). The ring-like area showed a gradual, faint enhancement and hyperintensity in the hepatobiliary phase, which is distinct from the surrounding hypointense tumoral components (arrow in G).

Both lesions in S5 and S8 showed hypointense on T1-weighted images (Figures 1A and 2A) and hyperintense on fat-suppressed T2-weighted images (Figures 1B and 2B). Diffusion-weighted imaging revealed significant hyperintensity within the lesions (Figures 1C and 2C), with corresponding low apparent diffusion coefficient values (0.65 × 10^−3^ mm^2^/s for the S5 lesion and 0.85 × 10^−3^ mm^2^/s for the S8 lesion) (Figures 1D and 2D), thereby indicating restricted diffusion. On gadoxetic acid-enhanced (EOB) MRI, the 2 lesions showed a faint enhancement in the arterial phase (Figures 1E and 2E) and were isointense relative to the surrounding liver parenchyma in the portal phase (Figures 1F and 2F) and showed hypointense in the hepatobiliary phase (Figures 1G and 2G). The peri-tumoral regions demonstrated early enhancement in the arterial phase (Figures 1E and 2E) with persistent enhancement in the portal phase (Figures 1F and 2F) and appeared isointense to the surrounding liver parenchyma in the hepatobiliary phase (Figures 1G and 2G). In the S5 lesion, a faint linear hyperintensity was observed adjacent to the lesion on diffusion-weighted imaging (Figure 2C), and this area showed enhancement in the arterial phase (Figure 2E) with persistent enhancement in the portal phase (Figure 2F). In addition, the lesion contained an internal ring-like area that appeared hypointense on T2-weighted images (Figure 2B). This area showed gradual faint enhancement and hyperintensity in the hepatobiliary phase, distinct from the surrounding hypointense tumoral components (Figure 2G).

## Treatment and pathological findings

The presence of malignant hepatic tumors, including hepatocellular carcinoma (HCC), could not be ruled out. Hence, a laparoscopic anterior sectionectomy of the liver was performed.

**Figure 3:**
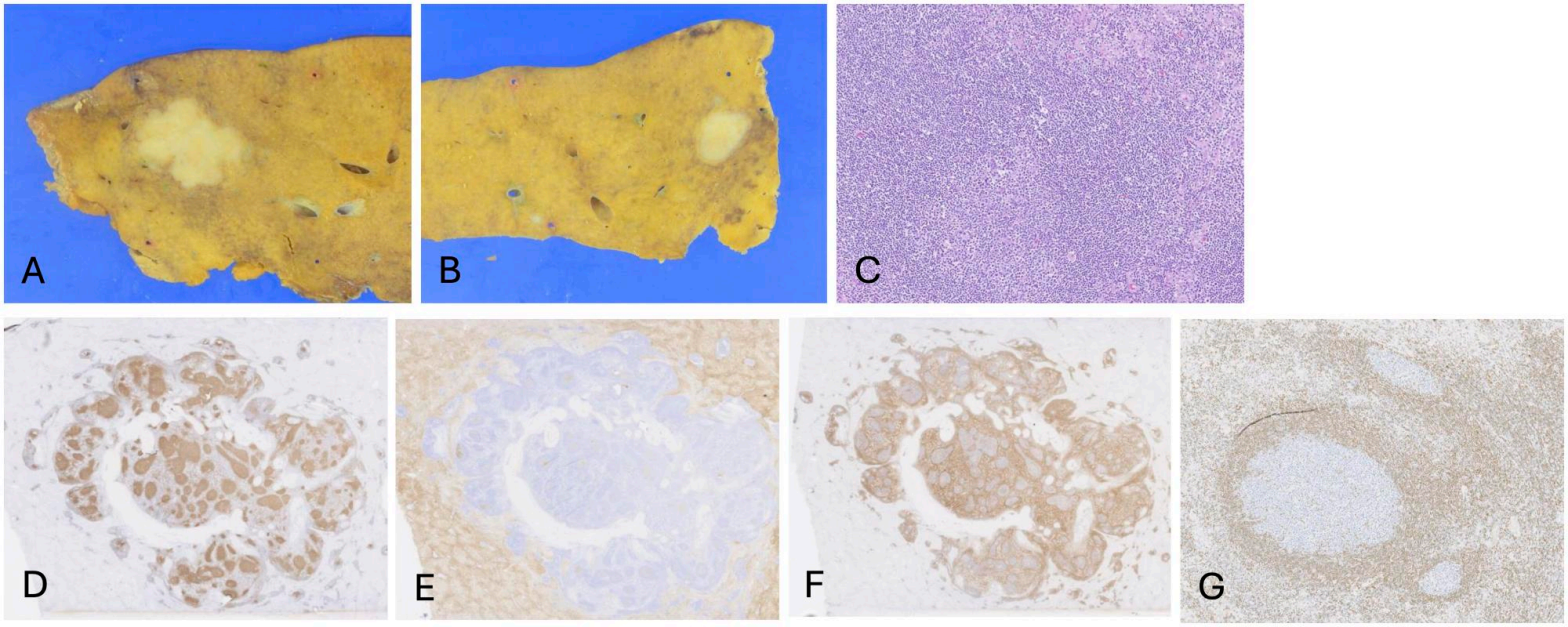
Pathological findings of hepatic pseudolymphoma. (A and B) Gross examination showed whitish, non-encapsulated lesions in the S5 (A) and S8 (B) tumors. (C) Hematoxylin and eosin staining showed that there was dense lymphoplasmacytic infiltration in the subserosa region of the liver (S5), forming various-sized lymphoid follicles with enlarged germinal centers. (D-G) Immunohistochemical staining showed CD20-positive B cells in the follicles (D), CD10-positive germinal centers (E), CD3-positive interfollicular T cells (F), and BCL-2-negative cells (G).

**Figure 4:**
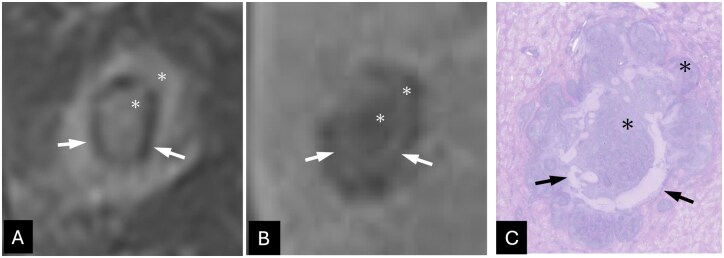
Radiologic–pathologic correlation of hepatic pseudolymphoma in segment 5. (A and B) The ring-like area within the S5 lesion appeared hypointense on fat-suppressed T2-weighted imaging (arrows in A) and hyperintense in the hepatobiliary phase (arrows in B). (C) Pathologically, this area corresponded to intratumoral fibrotic tissue (arrow in C). Inflammatory cell infiltration with lymphoid follicle formation was distributed peripheral to the fibrotic area (asterisks in A-C).

Gross examination of both the S5 and S8 tumors revealed whitish lesions without a fibrous capsule (Figures 3A and B). Histopathological analysis showed dense inflammatory cell infiltration with prominent reactive lymphoid follicle formation arranged in a concentric pattern (Figure 3C). In the S5 lesion, a ring-like fibrotic tissue was observed within the lesion, and inflammatory cell infiltration with lymphoid follicle formation was distributed peripheral to the fibrotic area (Figure 4). Significant lymphocytic infiltration was also noted in the portal tracts surrounding the lesions. Immunohistochemical staining showed CD20-positive B cells in the follicles (Figure 3D), CD10-positive germinal centers (Figure 3E), CD3-positive interfollicular T cells (Figure 3F), and BCL-2-negative cells (Figure 3G). Only a few IgG4-positive plasma cells were identified. Hence, IgG4-related disease was less likely. Based on these findings, a final diagnosis of hepatic pseudolymphoma (HPL) was established.

## Discussion

HPL, also known as reactive lymphoid hyperplasia of the liver, is a rare benign lymphoproliferative disorder characterized by polyclonal lymphoid follicles with reactive germinal centers. Hepatic involvement is rare and often detected incidentally.^1^ HPL predominantly affects women around the sixth decade. The lesions commonly measure ≤2 cm, and they are solitary. However, multiple lesions have been reported in approximately 20% of cases.^1^^,^^2^ Associations of the lesions with chronic liver disease and inflammatory/autoimmune conditions have been suggested.^1^ Overall, our patient—a woman in her 50 s with non-alcoholic fatty liver disease—had multiple relatively small hepatic lesions, which are consistent with the clinicodemographic features of HPL.

On MRI, HPL typically shows hypointense on T1-weighted images and hyperintense on T2-weighted images and restricted diffusion. Moreover, linear hyperintensity along the adjacent portal tract on diffusion-weighted imaging has been described as a characteristic imaging finding of HPL.^3^ Dynamic contrast-enhanced MRI often shows arterial-phase hyperenhancement with delayed hypointensity. Further, it may demonstrate perinodular or irregular peripheral enhancement. In the hepatobiliary phase of EOB MRI, lesions are usually significantly hypointense relative to the surrounding liver.^4^^,^^5^ In the current case, the 2 lesions essentially exhibited these typical imaging features. However, the S5 lesion also showed findings indicative of fibrosis, appearing hypointense on T2-weighted images and hyperintense in the hepatobiliary phase. Histopathological examination revealed reactive lymphoid follicular hyperplasia predominantly at the lesion periphery and focal intratumoral fibrosis forming a ring-like structure. This fibrotic component spatially corresponded to the area of relative hyperintensity in the hepatobiliary phase, indicating an imaging–pathologic correlation (Figure 4). Fibrotic areas generally appear hyperintense on hepatobiliary-phase images due to contrast retention and/or T1-shortening effects. Although stromal fibrosis has been reported in HPL,^4^ to the best of our knowledge, an intratumoral ring-shaped fibrosis has not been previously described.

According to the Liver Imaging Reporting and Data System version 2018, a targetoid appearance in the hepatobiliary phase, showing a relatively hypointense peripheral rim and central hyperintensity, is considered a feature suggestive of non-HCC malignancy and is believed to reflect peripheral hypercellularity with internal stromal fibrosis or ischemia.^6^ In contrast, the ring-like appearance in the present case pathologically corresponded to intratumoral ring-shaped fibrosis and reflected a mechanism distinct from that of the targetoid appearance. Recognition of this unusual imaging feature may help avoid misinterpretation as malignancy and improve diagnostic confidence in the evaluation of atypical hepatic lesions. Speckled enhancement in the hepatobiliary phase has been reported in primary hepatic mucosa-associated lymphoid tissue lymphoma, where it is attributed to residual hepatocytes within lymphoid infiltrates.^7^ However, in our case, the hyperintense foci corresponded to fibrosis rather than preserved hepatocytes.

In HCC with typical imaging features, a fibrous capsule or pseudocapsule is a well-recognized histopathologic feature and may account for a rim-like appearance on imaging.^8^ Variant tumors with prominent fibrosis are also common. Scirrhous-type HCC is characterized by abundant and diffuse intratumoral fibrosis.^9^ In addition, fibrolamellar carcinoma shows characteristic lamellar collagen bundles and frequently demonstrates a central scar.^10^ However, the discrete fibrotic ring observed in our case appears to differ from these tumors.

The current report is limited by its single-case design and further studies must be conducted to validate whether this imaging feature is a characteristic finding of HPL or merely an incidental phenomenon. Nevertheless, this case indicates that signal heterogeneity in the hepatobiliary phase of EOB MRI may reflect underlying pathological heterogeneity in HPL. Knowledge about this potential imaging–pathologic correlation may help prevent the misdiagnosis of such lesions as malignant disease.

## Learning points

- HPL predominantly affects women around the sixth decade and is often detected incidentally as a small (≤2 cm), solitary lesion; multiple lesions occur in approximately 20% of cases.
- Characteristic MRI findings of HPL include hyperintensity within the lesion and in the area along the adjacent portal tract on diffusion-weighted imaging, as well as arterial-phase hyperenhancement in the peritumoral adjacent area on dynamic contrast-enhanced MRI.
- In HPL, hepatobiliary-phase hyperintensity reflects fibrosis distinct from that seen in malignant hepatic tumors.
